# Maternal-Fetal Pharmacology of Drugs: A Review of Current Status of the Application of Physiologically Based Pharmacokinetic Models

**DOI:** 10.3389/fped.2021.733823

**Published:** 2021-11-03

**Authors:** Nupur Chaphekar, Prerna Dodeja, Imam H. Shaik, Steve Caritis, Raman Venkataramanan

**Affiliations:** ^1^Department of Pharmaceutical Sciences, School of Pharmacy, University of Pittsburgh, Pittsburgh, PA, United States; ^2^Department of Obstetrics, Gynecology and Reproductive Sciences, Magee Women's Hospital of UPMC, School of Medicine, University of Pittsburgh, Pittsburgh, PA, United States; ^3^Department of Pathology, School of Medicine, University of Pittsburgh, Pittsburgh, PA, United States

**Keywords:** maternal, fetal, pharmacology, pregnancy, PBPK

## Abstract

Pregnancy and the postpartum period are associated with several physiological changes that can alter the pharmacokinetics (PK) and pharmacodynamics (PD) of drugs. For certain drugs, dosing changes may be required during pregnancy and postpartum to achieve drug exposures comparable to what is observed in non-pregnant subjects. There is very limited data on fetal exposure of drugs during pregnancy, and neonatal exposure through transfer of drugs via human milk during breastfeeding. Very few systematic clinical pharmacology studies have been conducted in pregnant and postpartum women due to ethical issues, concern for the fetus safety as well as potential legal ramifications. Over the past several years, there has been an increase in the application of modeling and simulation approaches such as population PK (PopPK) and physiologically based PK (PBPK) modeling to provide guidance on drug dosing in those special patient populations. Population PK models rely on measured PK data, whereas physiologically based PK models incorporate physiological, preclinical, and clinical data into the model to predict drug exposure during pregnancy. These modeling strategies offer a promising approach to identify the drugs with PK changes during pregnancy to guide dose optimization in pregnancy, when there is lack of clinical data. PBPK modeling is also utilized to predict the fetal exposure of drugs and drug transfer via human milk following maternal exposure. This review focuses on the current status of the application of PBPK modeling to predict maternal and fetal exposure of drugs and thereby guide drug therapy during pregnancy.

Pregnant women take one to three medications on an average in addition to the routine iron and vitamin supplements recommended during pregnancy (1). Pregnant women take medications for acute illnesses such as nausea and vomiting, upper respiratory tract and urinary tract infections or for chronic conditions such as psychiatric disorders, HIV infection, epilepsy, organ transplantation, rheumatological conditions, or substance abuse disorder. Pharmacotherapy is also needed for pregnancy-induced conditions like hypertensive disorder, preterm labor and gestational diabetes (2). Pregnant women and their fetuses are orphan populations with regards to information on the safety and efficacy of drugs. Ninety eight percent of the drugs approved in the United States between 2000 and 2010 have insufficient data on drug dosing during pregnancy, while seventy percent of them have no data on drug use in pregnancy (3). Pregnant women are excluded from clinical studies due to ethical, fetal safety and medico-legal concerns. Therefore, there is limited data available on PK and PD of drugs used in pregnancy. Table 1 lists the issues and potential confounding factors contributing to the lack of PK and PD data in pregnancy. Current dosing recommendations in pregnancy are based on data obtained from non-pregnant population. In this context, modeling and simulation techniques like PopPK or PBPK can provide additional information regarding appropriate drug dosing in this special population. A summary of ideal studies that could be conducted during pregnancy and the next best alternative or alternate approaches that can be used when a clinical study is not practical to obtain necessary data, is presented in Table 2.

**Table 1:** Need for designed pharmacological studies performed during pregnancy, lactation and postpartum.

**Scope of the problem**	**Contributors to the problem**
• Inadequate pharmacological studies performed during pregnancy, lactation and postpartum • Limited data on pregnancy mediated changes in drug exposure and response • Optimal dosing for pregnant, lactating, postpartum women unclear for most medications • Impact of drug exposure on fetal growth and development is unclear for almost all medications used during pregnancy • Limited data on drug transfer through breast feeding • Limited incentive for industries (safety—liability issues)	• Pregnancy is an exclusion in most clinical trials • Inadequate funding for clinical pharmacology research in pregnant, lactating and postpartum women • Inadequate number of investigators qualified to perform or engaged in such studies • Inconvenient study designs for participants • Need for innovative sampling techniques and modeling approaches

**Table 2:** Ideal studies in pregnancy and alternative approaches.

**Ideal studies**	**Next best alternatives**	**Alternate approaches**
• Drug exposure studies (Pharmacokinetics over a dosing interval) in first, second, third trimester and post-partum • Drug response studies over a dosing interval (first, second, third trimester and post-partum) • Maternal drug safety assessments (first, second, third trimester and post-partum) • Fetal / Neonatal drug safety assessments (monitoring of neonates and newborn) • Drug excretion in breast milk (total amount excreted in breast milk over a dosing interval)	• Surrogate drug exposure studies (limited sampling strategy or trough level) in first, second, third trimesters and post-partum • Limited drug response studies (first, second, third trimester and post-partum) • Placental (*in vitro*) perfusion studies • Cord blood sampling for fetal exposure assessments • Milk to plasma ratio for drugs in lactating women • Placental perfusion studies • Placenta on a chip study	• Predictions based on probe drug studies for DME and transporters • Population PK modeling • PBPK modeling and simulations

## Physiological Changes During Pregnancy

Several physiological changes occur during pregnancy that help support the growth and development of the fetus. The absorption, distribution, metabolism and excretion processes of drugs can be altered during pregnancy and may contribute to altered PK of drugs. Table 3 summarizes pregnancy mediated physiological changes that can impact PK processes. Reduced gastrointestinal motility and delayed gastric emptying time during pregnancy can reduce drug absorption. There is an increase in gastric pH during pregnancy which can lead to changes in absorption of acidic drugs due to increased ionization (10, 11). A systematic study evaluating the impact of changes in drug absorption on pharmacokinetics after intravenous vs. oral administration during pregnancy and postpartum is lacking. Several physiological changes may alter drug distribution such as increased plasma volume, maternal plasma protein dilution or organ volume variation (fat) (12–14). The expression and activity of certain CYP enzymes change during pregnancy which may lead to change in metabolism of selected substrates. The metabolism of drugs catalyzed by cytochrome P450 (CYP) isoenzymes CYP3A4, CYP2D6, CYP2C9 and certain uridine glucuronosyltransferases (UGT) isoenzymes UGT1A4 and UGT1A9 is increased during pregnancy (15) and the metabolism of CYP1A2 and CYP2C19 substrates is decreased during pregnancy (15, 16).

**Table 3:** Physiological changes and potential impact on PK of drugs.

**Pharmacokinetic parameter**	**Effect of pregnancy**	**Potential impact on pharmacokinetics**	**Clinical example**
Absorption	Decrease in gastrointestinal motility and gastric emptying time Increase in gastric pH Increase in gastrointestinal blood flow Alterations in enzymes and transporters involved in absorption of drugs	Increase or decrease in the rate of absorption Increase or decrease in bioavailability	Aspirin C_max_ decreased by 29% during pregnancy (4) Lower C_max_ of metoprolol during pregnancy (5)
Distribution	Increase in cardiac output Increase in total body water and fat Decrease in plasma protein binding	Increase in volume of distribution	Increase in volume of distribution of metoprolol during pregnancy (5)
Metabolism	Alterations of CYP and UGT enzyme activity Increase in hepatic blood flow	Increase or decrease in metabolism of substrates	Decrease in clearance of caffeine (CYP1A2 substrate) during pregnancy (6) Increase in Clearance of lamotrigine (UGT1A4 substrate) during pregnancy as compared to postpartum (7)
Excretion	Increase in renal blood flow Increase in glomerular filtration rate Alterations of enzymes and transporters involved in tubular reabsorption and secretion	Increase in renal excretion Increase or decrease in tubular reabsorption and secretion	Unbound renal secretion of digoxin increased during pregnancy due to increased P-gP activity (8) Increased renal secretion and renal clearance of amoxicillin during pregnancy as compared to postpartum (9)

Accumulating *in-vivo* and *in-vitro* data suggests that the increased levels of steroid hormones during pregnancy might be responsible for altered metabolism of certain substrates (17). For example, UGT1A1 up-regulation was seen in progesterone treated HEPG2 cells co-transfected with PXR as compared to control cells. An increase in the glucuronidation (UGT1A4) was observed in 17-beta estradiol treated HEPG2 cells co-transfected with ERα receptor (18). Progesterone treatment caused up-regulation of UGT1A in pregnant humanized UGT1A/ PXR mice as opposed to pregnant humanized UGT1A mice with PXR knockout suggesting the role of PXR activation leading to the up-regulation of UGT1A enzymes (19). The renal excretion of drugs is increased during pregnancy due to a 60–80% increase in renal blood flow and a 50% increase in glomerular filtration rate (20). To date there is limited data available elucidating the effect of pregnancy on intestinal, hepatic and renal transporters involved in the absorption, distribution, efflux, secretion and reabsorption of drugs.

## PBPK Modeling to Predict Drug Exposure During Pregnancy

Model-based approaches can provide some information regarding drug exposure and drug dosing in various patient populations when direct clinical data is not available. PBPK is a tool that can be used to predict drug exposure in such patient populations. This model-predicted data can be used to optimize drug dosing in special patient populations and can be further fine-tuned as more clinical data becomes available.

**Figure 1:**
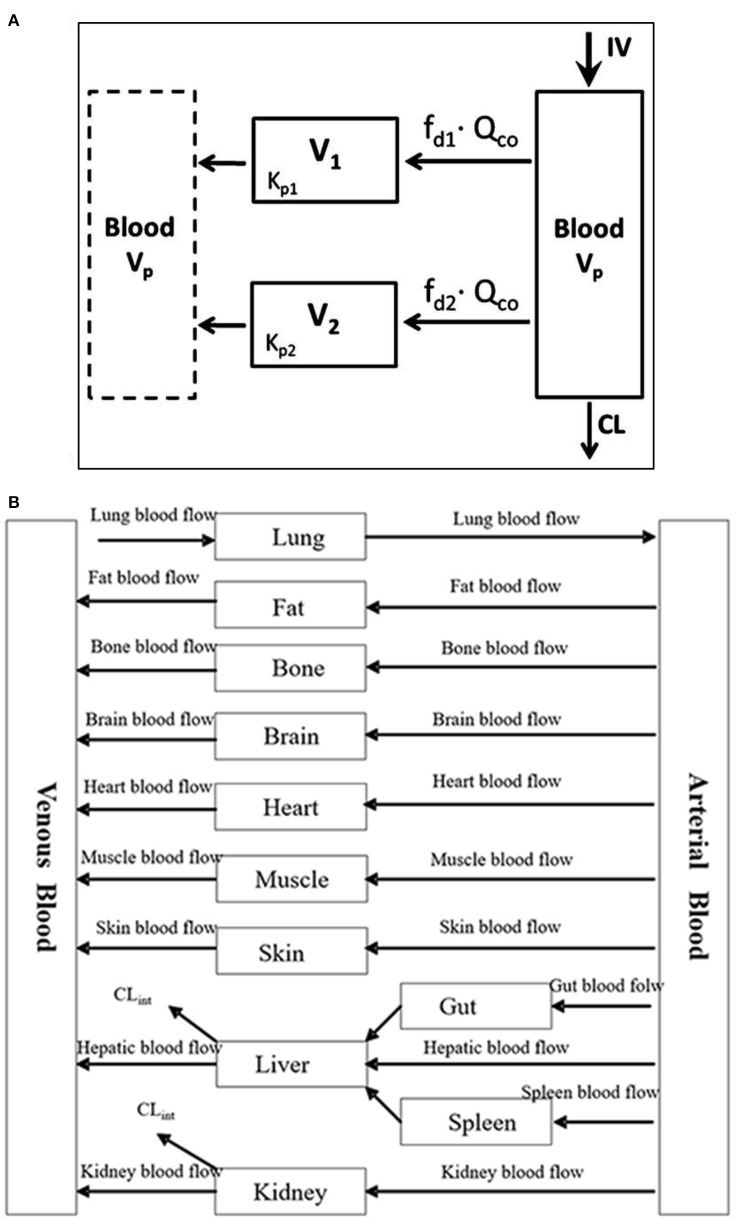
**(A)** Minimal PBPK model with two tissue compartments (26). **(B)** Example of a PBPK model (27).

**Figure 2:**
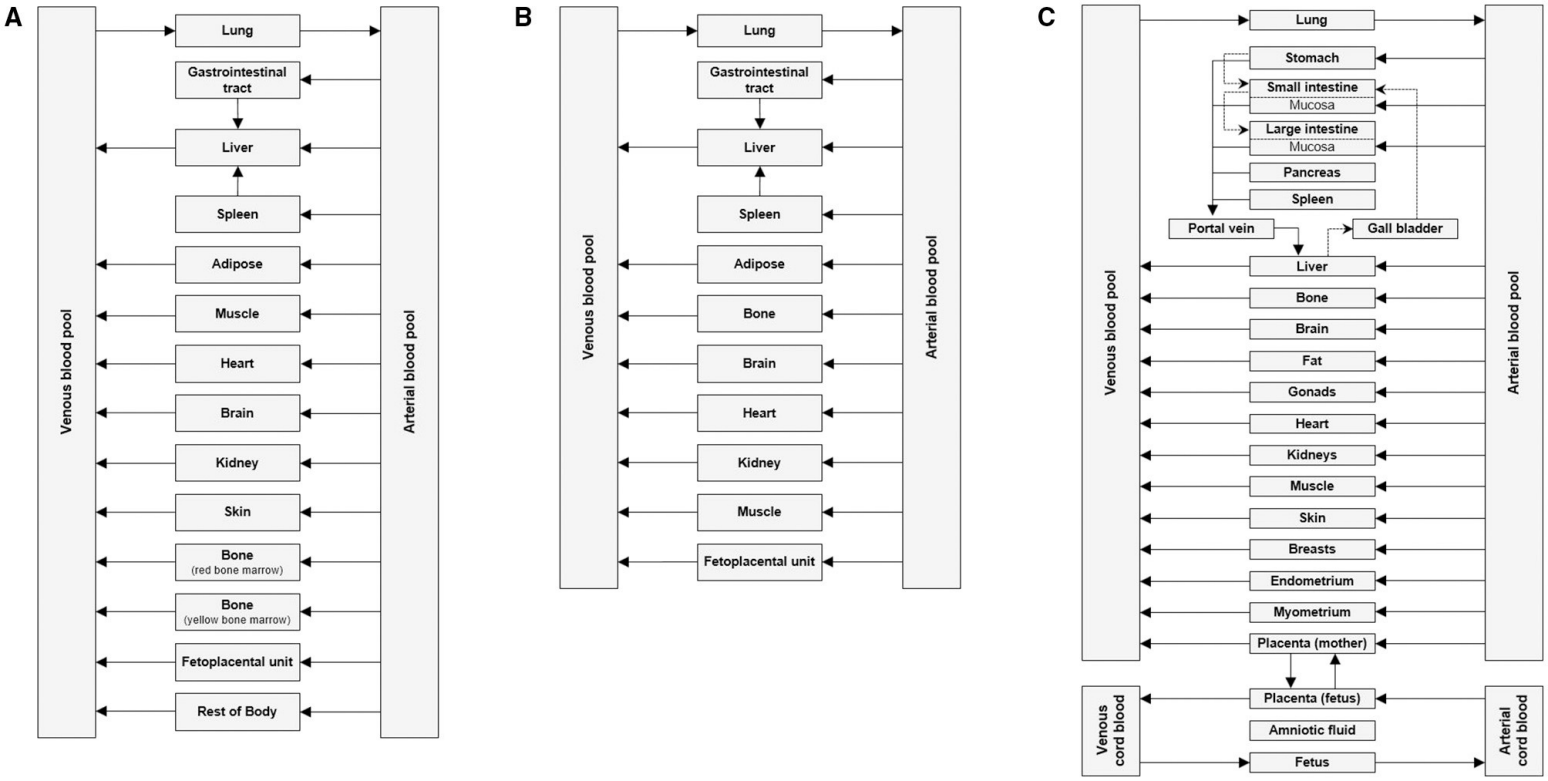
Basic structure of p-PBPK model in **(A)** Gatsroplus **(B)** SimCyp and **(C)** Open Systems Pharmacology (28).

PBPK is a mechanistic approach that has been used in the drug development processes to determine safe and optimal doses to be used in clinical trials, estimate drug exposure in special populations and also to predict drug-drug interactions (21). It can be used as a viable alternative to generate clinical data in special patient populations. Regulatory agencies such as the US FDA and the European Medicines Agency have accepted the use of PBPK modeling to facilitate the decision-making process for conducting a clinical study in submissions for Investigational New Drug and New Drug Applications (22–24). PBPK models are multicompartmental models in which each compartment corresponds to one or more organ and is interconnected by the circulatory system. It integrates important physiological parameters (e.g., blood flow, enzyme and transporter abundance, cardiac output, glomerular filtration rate) and drug related parameters (blood-to-plasma ratio, plasma protein binding, permeability, solubility, *in vitro* metabolism or transport) which are known to influence drug PK and PD (25). Figure 1A represents an example of a minimal PBPK model (26) and Figure 1B represents an example of a PBPK model with each tissue/organ in the body being considered as a separate compartment (27). Pregnancy creates the need for additional compartments in the PBPK model. Figure 2 depicts the structure of pregnancy-PBPK (p-PBPK) model used in three different PBPK modeling software. The most important compartment in a p-PBPK model is the fetal unit. This is combined into a single “lumped” compartment known as the fetoplacental unit in the Simcyp and GastroPlus software. The fetoplacental unit incorporates the fetus, placenta, amniotic fluid, membranes and umbilical cord as depicted in Figures 2A,B. However, in the Open Systems Pharmacology software package, each of these units are considered discrete and accounted for separately, along with the inclusion of myometrium and endometrium, as seen in Figure 2C. Table 4 summarizes the physiological parameters that are considered in the Simcyp p-PBPK model.

**Table 4:** Physiological parameters that are modified for pregnancy prediction in Simcyp p-PBPK model.

**List of parameters**
Cardiac output Total body weight Total fat Plasma volume Red blood cell volume Hematocrit Serum albumin Skin blood flow rate Adipose blood flow rate Renal blood flow rate Fetoplacental unit blood flow rate Enzyme and transporter activity

## Current Status of Pregnancy PBPK Models

Physiological changes during pregnancy are gestational age dependent. For example, the activity of UGT1A4 increased by 200% during the first and second trimesters and by 300% during the third trimester leading to increased clearance of lamotrigine (7). Similarly, the changes in organ blood flow, activity of certain metabolic enzymes and transporters are dependent on gestational age. The p-PBPK models incorporate these gestational age-related physiological changes into a normal PBPK model to simulate pregnant population. These p-PBPK models can then be used to predict the gestational age dependent pharmacokinetics of different drugs.

Several p-PBPK models have been developed and evaluated for antiretroviral, anti-malarial, psychoactive drugs, drugs used for the treatment of substance use disorder and environmental chemicals. Although these models have been able to predict the pharmacokinetics of certain drugs during pregnancy reasonably well, there are still several challenges that remain unresolved. There is no/limited information available to fully evaluate all the assumptions that are used in such models. There is paucity of data on combined effect of pregnancy and disease state (e.g., diabetes, malaria, hypertensive disorder) on gestational age-related changes in various physiological parameters and hence the predictions must be interpreted with caution. Data for drug elimination kinetics are typically scaled from *in-vitro* cell culture experiments and these experiments do not account for all the physiological changes which necessitates additional extrapolation factors to be incorporated. There is lack of information regarding changes in all drug metabolizing enzyme and transporter activity across gestational ages. Enzyme or transporter activity determined using probe drug data is specific to the trimester in which the study was conducted and cannot be extrapolated to other trimesters.

An exhaustive literature search was conducted using PubMed with the keywords *PBPK and pregnancy*. The results from the search with clinical observations are listed in Table 5 with specific examples discussed below.

**Table 5:** Review of published p-PBPK models.

**Compound**	**Route of administration**	**Clinical observations**	**Recommended dose adjustment based on PBPK modeling**	**Software**	**Reference**
Acetaminophen	IV and oral dosing	Lower acetaminophen concentrations during pregnancy as compared to non-pregnant women	No dose adjustments since there is lack of data on toxicity of the metabolite NAPQI	Open Systems Pharmacology®	(29)
Amoxicillin	IV bolus and infusion	Increased renal clearance during pregnancy and postpartum	May need increased dosing No clinical recommendations	Open Systems Pharmacology®	(30)
Betamethasone	IV, IM and oral dosing	Increased clearance during pregnancy	No clinical recommendations	Simcyp®	(31)
Buprenorphine	Sublingual	Decreased buprenorphine exposure during pregnancy as compared to postpartum	Increased dose/ more frequent dosing	Simcyp	(32)
Caffeine	Oral dosing	Increased maternal and fetal exposure during pregnancy due to reduced CYP1A2 activity	Limit caffeine intake	GastroPlus®	(33)
Caffeine, Midazolam, Nifedipine, Metoprolol Ondansetron, Granisetron, Diazepam and Metronidazole	IV and oral dosing	Increase in clearance of CYP2A6, CYP2E1, CYP2D6 and CYP3A4 substrates and decreased clearance of CYP1A2 and CYP2C19 substrates	Likely changes in dosing No clinical recommendations	Open Systems Pharmacology®	(34)
Caffeine, Metoprolol, Midazolam	IV Bolus, Oral dosing	100% increase, 30% decrease and a 35% decrease in the exposure of caffeine, metoprolol, and midazolam respectively during pregnancy	Decreased dose for caffeine and increased dose for metoprolol and midazolam	Simcyp®	(35)
Cefazolin, Cefuroxime, Cefradine	IV and oral dosing	Increased clearance of the three drugs during pregnancy	Increased dose during pregnancy	Open Systems Pharmacology®	(36)
Ceftazidime, Cefuroxime, Fluconazole, Aztreonam, Imipenem, Ceftriaxone	IV and Oral dosing	Decrease of *in vivo* drug exposure (for all 6 drugs) in pregnant women due to increased renal clearance	No dose changes	Simcyp®	(37)
Cefuroxime, Cefazoline	IV infusion, IV bolus or infusion	Model accurately predicts changes in renal clearance for both drugs, however inclusion of postpartum data is necessary for fine tuning	No clinical recommendations	GastroPlus®	(38)
Darunavir boosted with ritonavir	Oral dosing	Decreased Darunavir exposure during second and third trimester of pregnancy	Increased dose or dosing frequency during pregnancy	Simcyp®	(39)
Dolutegravir	Oral dosing	Dose of 50 mg q.d Dolutegravir provides sufficient fetal exposure, resulting in 90% viral inhibition	No dose changes	Berkeley Madonna	(40)
Dolutegravir, Raltegravir	Oral dosing	Decreased exposure during pregnancy	No dose changes	Open Systems Pharmacology®	(41)
Emtricitabine and Acyclovir	Oral dosing	Lower emtricitabine and acyclovir concentrations during pregnancy with the lowest concentrations during the third trimester	No dose changes	Open Systems Pharmacology®	(42)
Emtricitabine, Dolutegravir, Raltegravir	Oral dosing	Neonatal washout kinetics observed for all three drugs	No clinical recommendations	Open Systems Pharmacology®	(43)
Indomethacin	Oral dosing	Higher indomethacin clearance during second trimester as compared to non-pregnant women.	Higher dosing requirement during pregnancy	Gastroplus®	(44)
Indomethacin	Oral dosing	Decrease in indomethacin exposure by 14, 24, and 32% in the first, second and third trimester respectively, compared to non-pregnant women.	Additional clinical studies warranted to provide optimal dosing recommendations	Simcyp®	(45)
Metformin, digoxin, emtricitabine, midazolam	Oral dosing	Decreased exposure during pregnancy due to increased clearance	No clinical recommendations	GastroPlus®	(46)
Metformin, Tacrolimus, Oseltamivir	Oral dosing	Increased renal clearance of metformin during pregnancy as compared to postpartum. 20 % decrease in AUC of tacrolimus between 1^st^ and 3^rd^ trimester. AUC of parent drug (oseltamivir) similar but AUC of metabolite (oseltamivir carboxylate) 30% lower during pregnancy.	No clinical recommendations	Simcyp®	(47)
Methadone, Glyburide, Phenytoin	Oral dosing	Increased clearance of methadone and glyburide during pregnancy as compared to postpartum	No clinical recommendations	Simcyp®	(48)
Midazolam, Nifedipine, Indinavir	Oral dosing	Increased clearance during pregnancy	No clinical recommendations	MATLAB	(49)
Midazolam, Theophylline, Zidovudine, Nevirapine, Emtricitabine, Lamivudine, Ondansetron, Diazepam, Metronidazole, Cefuroxime	IV and oral dosing	Increase in fetal exposure with pregnancy age for all drugs	No clinical recommendations	GNU MCSim	(50)
Piperaquine	Oral dosing	Pharmacokinetics unchanged as compared to non-pregnant women	No need for dosage adjustment	Simcyp®	(51)
Quetiapine	Oral dosing	Decreased concentrations during pregnancy	Dose increase during pregnancy	Simcyp®	(52)
Quetiapine, Aripiprazole	Oral and IV dosing	Progressively decreased plasma concentrations throughout pregnancy	Dose for both drugs needs to be increased in the second and third trimesters.	Open Systems Pharmacology®	(53)
Tenofovir, emtricitabine, lamivudine	IV and Oral dosing	Increase in renal clearance of drugs during pregnancy	No need for dosage adjustment	Simcyp®	(54)
Theophylline, Paroxetine, Clonidine, Dextromethorphan	Oral dosing	Increased concentration of theophylline during third trimester. 100–200% induction of CYP2D6 during third trimester adequately describes the pharmacokinetics of paroxetine, clonidine and dextromethorphan during pregnancy.	No clinical recommendations	Simcyp®	(55)
Ziprasidone	Oral dosing	No significant difference in exposure as compared to non-pregnant women	No dose adjustment necessary	Simcyp®	(56)

## Review of Pregnancy PBPK Models Reported in the Literature

PBPK modeling has been used as a tool to guide and optimize drug dosing in pregnancy for several drugs and scenarios discussed below.

### Drug Based Studies

Ziprasidone is an antipsychotic drug used to treat schizophrenia and other psychiatric disorders. It is administered orally and is metabolized by CYP3A4 primarily in the liver. Biesdorf et al. established a PBPK model to predict drug exposure during pregnancy using the Simcyp inbuilt pregnancy population which includes gestational age-related changes in blood flow, glomerular filtration rate, plasma protein binding etc. Since the model used the pregnancy population in Simcyp, some physiological changes that were not very specific to the route of elimination of Ziprasidone were also incorporated. The model predicted exposures correlated well with the clinical data and exposure of ziprasidone during pregnancy at 6, 20, and 34 weeks of gestation. Since the exposure of ziprasidone during pregnancy was comparable to non-pregnant women, no dose adjustment is recommended during pregnancy for this drug (56). Ke et al. developed a PBPK model to evaluate maternal exposure of the antenatal corticosteroids dexamethasone and betamethasone which are primarily metabolized by CYP3A4. In this model, the fraction of dexamethasone metabolized by CYP3A4 was obtained from a clinical DDI study with itraconazole. However, for betamethasone, an *in-vitro* study was conducted to investigate the role of CYP3A4 in its metabolism. Ideally a clinical DDI study should be conducted to verify the fraction of betamethasone metabolized by CYP3A4 (31).

Quetiapine, an antipsychotic drug metabolized mainly by CYP3A4 and CYP2D6, shows decreased exposure during pregnancy possibly due to known increase in the activity of these two enzymes. A PBPK modeling approach was used to optimize the dosing regimen to target a predetermined therapeutic range (52). Though the model recommended a dose increase during pregnancy, information about the pregnancy mediated changes on PD is also needed to implement the recommended change in the dose during pregnancy.

### Probe Drug-Based Studies

PBPK models developed previously have been also modified/ refined to determine the exposure of substrates during pregnancy. Ke et al. refined a previously published PBPK model to include CYP3A4 activity changes during the third trimester based on data from the probe drug midazolam and used it to predict the exposures of nifedipine and indinavir in pregnancy. The site of CYP3A4 induction during the third trimester was proposed to be mainly the liver (49). However, subsequent models were not able to reproduce these findings. The model by De Sousa Mendes *et al* showed that a 90–100% CYP3A4 induction is required to capture the PK changes in third trimester for drugs metabolized by CYP3A4 (57). Whereas the model by Dallmann et al. using Open Systems Pharmacology suggested that a 60% induction in liver and intestine CYP3A4 is enough to describe the observed PK changes (58). There is still ongoing discussion regarding the magnitude and site of CYP3A4 induction in pregnancy and there are several shortcomings with using probe drug data for CYP3A4 assessments of CYP3A activity for other drugs. The models developed cannot be applied to predict the pharmacokinetics of other drugs and also for evaluating the pharmacokinetics across different trimesters.

### Renally Cleared Drugs

Liu et al. developed a p-PBPK model for emtricitabine and acyclovir which are antiviral drugs primarily excreted unchanged in the urine by glomerular filtration and tubular secretion (42). The model had several limitations such as not accounting for potential changes in gastrointestinal absorption due to pregnancy. Additionally, since intravenous data for acyclovir in women was not available, observed drug concentrations were extrapolated based on PK data from men. A previously developed PopPK model for ganciclovir, a drug in the same class as acyclovir, has shown higher ganciclovir clearance in women than men after correcting for individual body surface area and glomerular filtration (59). Therefore, it is likely that there may be a significant underestimation of acyclovir as well in the model developed by Liu et al.

### Pregnancy and Genotype Impact

A limited number of PBPK models in the literature have evaluated the impact of genotype on pharmacokinetics of drugs during pregnancy. Efavirenz which is used for the treatment of human immunodeficiency virus (HIV) is metabolized by the highly polymorphic enzyme CYP2B6. Though both 400 mg and 600 mg doses show similar efficacy, a 400 mg dose is suggested to avoid dose related toxicities. However, there is limited data on the PK of 400 mg dose in pregnancy. p-PBPK model developed by Chetty et al. using Simcyp evaluated the pharmacokinetics after a reduced dose of 400 mg in CYP2B6 extensive metabolizers. The model predicted that approximately 57% of extensive metabolizers would show trough concentrations below the therapeutic target during third trimester, suggesting dose reduction during pregnancy may lead to therapeutic failure in extensive metabolizers (60). The utility of this model to predict drug exposure in rapid and ultra-rapid metabolizers during pregnancy remains unknown. Additionally, evidence suggests that race and ethnicity have an impact on CYP2B6 activity. The model by Chetty et al. has been developed and evaluated only for the Caucasian population and therefore the generalizability of the model to other populations is questionable. Models incorporating other inbuilt populations such as in Simcyp (e.g., Japanese, Chinese etc.) can be used to optimize drug dosing in the non-Caucasian populations (61).

### Pregnancy and Drug Response

p-PBPK models have also been extended to determine the PD effect of drugs used in pregnancy. Darakjian et al. developed a PBPK-PD model for caffeine in pregnancy. The PD model evaluated the effect of caffeine on phosphodiesterase enzyme (PE), cyclic adenosine monophosphate (cAMP) and epinephrine levels, which are factors associated with increased miscarriage risk. Increased caffeine plasma levels due to reduction in CYP1A2 activity during pregnancy led to greater inhibition of the PE enzyme, higher cAMP and greater increase of epinephrine levels which could increase the risk of pregnancy loss. Despite not being validated, the model was able to predict the increased concentration of caffeine in the fetoplacental compartment indicating its potential utility (33). Alqahtani et al. developed a PBPK-PD model to estimate concentrations of indomethacin in the second trimester of pregnancy and to support dose adjustment based on PD rationale in the pregnant population. Although the PBPK-PD model suggested a higher indomethacin dosing requirement during pregnancy, it cannot be directly used in clinical practice without further *in-vivo* validation (44).

### Pregnancy and Drug Interactions

PBPK models can be potentially used to predict drug-drug interactions in pregnancy when it is difficult to conduct clinical studies in vulnerable populations. Piperaquine is an antimalarial drug used during pregnancy. Approximately 1 million pregnancies in sub-Saharan Africa are complicated with co-infection of human immunodeficiency virus (HIV) and malaria, however there is paucity of data on anti-HIV medication mediated exposure changes of piperaquine during pregnancy (62). Olafuyi et al. developed a PBPK model to predict the drug-drug interaction potential between piperaquine and anti-HIV drugs (ritonavir and efavirenz) for Thai, Papua New Guinean, and Sudanese populations. The model showed no change in piperaquine PK due to co-administration of anti-HIV drugs and indicated no need for a change in the dose (51).

## Current Status of p-PBPK Models Used to Determine Fetal Exposure

The p-PBPK model becomes more complex upon addition of the fetoplacental unit since the model requires inclusion of placental transfer parameters, fetus and placental enzyme and transporter kinetics and blood flow to various additional anatomical units to predict exposure in fetus.

The *in-vitro* placental perfusion model is one of the tools used to study transplacental transfer of drugs (63). It can also be used to investigate the effect of exogenous and endogenous chemicals on maternal and fetal perfusion and transfer. It offers several advantages as the placental barrier is maintained and separate perfusion of the maternal and fetal side can be achieved. However, information about transplacental drug transfer and expression of enzymes and transporters during different stages of pregnancy cannot be obtained as the tissue for perfusion studies is normally available only at the time of delivery. The placenta is in a metabolically static state during these experiments as compared to the metabolically changing state during different stages of pregnancy (63, 64). Transplacental transfer parameters like diffusion, clearance index, elimination constant and placenta partition coefficient can be obtained from these experiments and incorporated in a PBPK model to predict fetal exposure later in pregnancy. The placental perfusion has been instrumental in developing PBPK models and has been used for predicting fetal exposure of dolutegravir, tenofovir, emtricitabine, and nevirapine (40, 57, 65).

Another approach is to incorporate data from *in-vitro* experiments using placental tissue, microsomes or human placental cell lines. Mian et al. developed a PBPK model to predict fetal exposure of acetaminophen. Different methods to estimate the placental transfer (ex *vivo* cotyledon perfusion experiments or scaling based on Caco-2 cell permeability experiments, physicochemical properties in MoBi) were incorporated in the model and the predictions show a comparable fetal exposure. Maturation of enzymes in the fetal liver was accounted for to determine the molar dose fraction of acetaminophen converted to N-acetyl-p-benzoquinone imine. The model incorporating the ex-*vivo* perfusion model data showed the best correlation with observed cord blood data for acetaminophen but may not hold true for all compounds (29). There is limited information available on placental enzymes and transporters in particular at various stages of pregnancy and further studies in this area would be helpful in developing IVIVE for placental clearance across various trimesters. Data obtained from primary placental cells, human choriocarcinoma cells or placenta-on-a-chip model may be more physiologically relevant to obtain transplacental parameters (66, 67). Protein abundance information for placental transporters which is available from recent reports can be incorporated into maternal-fetal PBPK models to further improve the model predictions (68).

Animal models offer another promising approach but differences in hemodynamics and placental structure can pose challenges in extrapolation of animal data to humans. The gestational age and the associated changes in physiology differ substantially between animals and humans requiring correction factors while extrapolating these data to humans. A PBPK model to predict fetal exposure of a brominated flame retardant, BDE-47 was developed by parameterizing the model with concentrations of BDE-47 from the literature and previous pharmacokinetic and toxicokinetic studies. This model was able to predict the fetal concentrations of BDE-47 in rats after maternal exposure within one standard deviation of the experimental data indicating its potential to be extrapolated to other species including humans after careful consideration of anatomical and physiological differences in placental structure and function (69).

Abduljalil et al. reviewed the literature for studies evaluating changes in fetal parameters (e. g., body weight, body surface area, body water, abdominal circumference, body fat) during fetal growth. This data was used to create mathematical algorithms to describe changes in these fetal parameters with gestational age which can potentially be added to the fetal PBPK model (70).

Transplacental transfer parameters from *in silico* models, *in vitro* and *ex vivo* studies have been incorporated into p-PBPK models. Codaccioni et al. developed p-PBPK model for ten compounds using four different models of placental exchange based on *in vitro, ex vivo*, and *in silico* information. The non-pregnant and pregnant as well as fetal PK simulations were compared with observed profiles at delivery for each of the ten compounds. A comparison of the model predictions across different trimesters of pregnancy yielded inconclusive results. These models can be optimized and potentially be used based on the purpose of the study and type of data and resources available (50). In the absence of clinical data to evaluate the fetal PBPK models, umbilical cord concentrations observed at delivery were used. Zhang et al. developed a maternal-fetal PBPK model which incorporated gestational age-related changes in fetal physiologic parameters such as fetal serum albumin, liver volume, uterus blood flow etc. Sensitivity analysis identified that a single time-point umbilical venous/ maternal plasma ratio is not reflective of the fetal exposure (71). The various gaps in knowledge for modeling maternal-fetal pharmacology are summarized in Table 6.

**Table 6:** Current gaps in modeling maternal-fetal pharmacology.

**Maternal pharmacology**	**Fetal pharmacology**
1. Lack of data on time course of changes in expression and activities of various phase 1 and 2 enzymes during pregnancy and postpartum 2. Lack of data on Time course of changes in various transporters during pregnancy and postpartum 3. Lack of data from same person during and post-delivery 4. Lack of PD measures—Relationship between exposure and response 5. Lack of information on potential impact of other comorbid conditions on PK/PD 6. Lack of PBPK models of biologics	1. Actual fetal exposure / blood and tissue concentration prediction not available—need for validation with meaningful clinical data 2. Lack of data on exposure response relationship in fetus 3. Placental enzymes and transporter expression data to incorporate transplacental transfer in PBPK model 4. Maternal-placental-fetal drug partitioning—factors impacting this such as plasma protein binding in mother, fetus, and role of placental transporters

An exhaustive literature search was conducted using PubMed with the keywords *PBPK and fetal exposure*. The publications from the search describing the development and validation of p-PBPK models to determine fetal exposure are presented in Table 7.

**Table 7:** List of published p-PBPK models to predict fetal exposure.

**Compound**	**Species in which model was developed and validated**	**Software**	**References**
Darunavir	Humans	Simcyp®	(72)
Dolutegravir	Humans	Berkeley Madonna	(40)
Dolutegravir	Humans-neonates	SimBiology®	(73)
Zidovudine, Theophylline	Humans	Simcyp®/ Matlab	(74)
Acetaminophen	Humans	Open Systems Pharmacology®	(75)
Nevirapine	Humans	R	(57)
Tenofovir, emtricitabine	Humans	Simcyp®, R	(65)
BDE-47 (polybrominated diphenyl ether)	Male, female (pregnant and non-pregnant rats)	ACSL® (Advanced Continuous Simulation Language)	(69)
Bisphenol A	Humans	R	(76)
Perfluorooctanoic acid (PFOA) and Perfluorooctane sulfate (PFOS)	Humans	ACSL® (Advanced Continuous Simulation Language)	(77)
Manganese	Humans	ACSL® (Advanced Continuous Simulation Language)	(78)
Thalidomide, Efavirenz	Humans	Simbiology®	(79)
Napthalene	Humans	CFD-PBPK	(80)
2,3,7,8-Tetrachlorodibenzo-p-dioxin (TCDD)	Pregnant female rats	ACSL® (Advanced Continuous Simulation Language)	(81)

## Examples of p-PBPK Models to Predict Fetal Exposure of Drugs

Fetal drug exposure is normally important from a fetal safety perspective. From efficacy point of view while normally one is interested in maternal drug exposure, there are conditions where fetal exposure is also important to maximize efficacy. Darunavir, an anti-HIV drug, primarily metabolized by CYP3A4 is routinely administered with CYP3A4 inhibitor ritonavir to maintain higher plasma concentrations during pregnancy. p-PBPK model was developed by incorporating information from *ex vivo* human placental perfusion studies to simulate fetal exposure after different dosing regimens (72). The model was validated by comparing maternal, fetal and amniotic fluid concentrations. The fetal concentration was compared with the single time-point umbilical cord concentration obtained at delivery. The model was able to capture the observed clinical data thus indicating that the placental perfusion data can be successfully integrated into p-PBPK models to predict fetal blood concentration at term. This approach is especially beneficial in the case of anti-HIV drugs to ensure that the half-maximal effective concentration is achieved in the fetus and the mother.

Zhang et al. developed a model to predict the placental transfer of passively diffusing drugs. The transplacental transfer parameters for zidovudine and theophylline were obtained using midazolam as the calibrator. The model was validated using single time-point maternal plasma and umbilical cord concentrations and the model was able to successfully predict the concentrations observed in patients. However, this model can only be used for drugs that undergo passive diffusion across the placenta. The use of a more sensitive calibrator that can predict placental transfer of a wide range of drugs with different physiochemical properties can enhance the utility of this model to predict fetal exposure of other drugs (82).

PBPK modeling has also been used to predict the fetal exposure to environmental chemicals (83). Bisphenol A (BPA) is an environmental chemical ingested through dietary and non-dietary sources. It is rapidly converted to nontoxic conjugates BPA-glucuronide (BPAG) and BPA-sulfate (BPAS) *via* glucuronidation and sulfation pathways. Sharma et al. developed a PBPK model for predicting the fetal exposure of bisphenol A which was evaluated against the observed BPA concentrations in cord blood, fetus liver and amniotic fluid following exposure from maternal blood (76). Parametrization of glucuronidation in fetus was done by scaling of *in-vitro* adult hepatocyte data in the absence of data from fetal hepatocytes which could have been a valuable addition to the model. Additionally, incorporating information on conjugation and deconjugation of BPA in placenta and fetus could lead to better prediction of the fetal exposure using this model.

Physiologically based toxicokinetic (PBTK) models are mathematical models that integrate absorption, distribution, metabolism and excretion processes for chemicals in biological systems. These models can serve as a tool to inform health risk assessments. They are traditionally based on extrapolating simulations in animal model to predict human exposure. For instance, Gingrich et al. developed a pregnancy specific p-PBTK model to predict bisphenol A and bisphenol S exposures in fetus (84). The model was calibrated using pregnant sheep data and results were extrapolated to assess the risk in humans However, this latter step remains uncertain due to major differences in placental physiology and structure between the species. More recently, high throughput toxicokinetic (HTTK) modeling has been used as an alternative in which models are parametrized with *in vitro* data, structure-derived physicochemical properties (e.g., QSAR) or species specific physiological data for several chemicals (85).

### PBPK Modeling to Predict Drug Exposure in Neonates

Bunglawala et al. built a neonatal PBPK model for dolutegravir using pediatric clinical data with assumptions that solubility, body composition and transporter expression were similar to adults (73). However, development and age-related changes are known but were not accounted in the model. Further, the possibility of drug exposure through maternal breast milk or placenta was not considered, though it is known that dolutegravir readily crosses the placenta.

In contrast to the approach described above, Liu et al. developed a PBPK model to link prenatal and postnatal pharmacokinetics using previously published p-PBPK models for emtricitabine, dolutegravir and raltegravir (43). The total drug amounts in fetal compartments at term delivery were predicted and incorporated as initial conditions in the neonatal PBPK model to predict drug concentrations in neonatal elimination phase after birth. Emtricitabine is eliminated unchanged in the urine by glomerular filtration and active tubular secretion mediated by Organic Cation Transporter 2 (OCT2). The OCT2 ontogeny applied in this model is based on data obtained from one term newborn only (86). Hence, additional *in-vitro* and clinical data are needed to further incorporate the ontogeny of OCT2 in the neonatal PBPK models. Additionally, the model should be tested and verified with information from other compounds as well as coupled maternal-fetal-neonatal PBPK models to understand early neonatal pharmacokinetics.

### PBPK Modeling to Predict Transfer of Drugs Through Human Milk and Infant Exposure

Maternal milk is a rich source of nourishment and breast-feeding is encouraged by the U.S. Department of Health and Human Services due to the beneficial effects for the mother as well as the infant. Maternal factors such as age, parity, breastfeeding patterns, milk composition and volume and physicochemical properties of the drug such as protein binding, molecular weight and lipophilicity affect the amount of drug transferred into human milk. Clinical studies focusing on quantifying the human milk exposure of drugs are needed. In the absence of clinical data, PBPK models have attempted to quantify infant exposure through human milk by integrating a breast tissue compartment. Loccisano et al. successfully developed a PBPK model to determine exposure of PFOA and PFOS in fetus and in infant through milk by extrapolating a previously developed and evaluated model in rats (77). As additional information on drug elimination kinetics in fetus and infant becomes available, it could be incorporated in to the model for better prediction of drug exposure in neonates (87).

Two differing approaches implemented in the prediction of infant exposure using PBPK are based on the method of drug uptake into human milk from plasma. One approach considers diffusion from drug in plasma via the breast tissue as done in PBPK modeling for lactational transfer of methylmercury (88). The other approach considers the direct passage of drug into the breast milk without considering the breast tissue as in the PBPK model to determine the infant exposure of organic pollutants (89).

Merrill et al. developed a PBPK model to predict perchlorate and iodide kinetics and subsequent perchlorate induced inhibition of iodide uptake in lactating mothers. The model was parameterized using data from previous models in male rat, lactating rat and non-pregnant women. However, this model has not been evaluated for perchlorate kinetics in humans due to lack of available clinical data (90). Isoniazid exposure to infant through breast milk was predicted using a validated PBPK model which accounted for the polymorphic expression of isoniazid metabolizing enzyme, N-acetyltransferase 2 (fast and slow metabolizers). Drug exposure was highest in slow metabolizing infants of slow metabolizing mothers, but the observed levels were still less than the infant exposure limit which is 10% of the maternal dose. The model was developed using information from ICRP reports which are generated based on data mainly from Caucasian population and should be cautiously extrapolated to other populations (82).

## Conclusions

There has been tremendous progress over the past few years in the use of PBPK modeling to predict maternal and fetal exposure of drugs. By integrating physiological data, preclinical data, and clinical data, PBPK can be used to predict maternal and fetal exposure and guide optimization of maternal dosing during pregnancy when pharmacokinetic studies cannot be readily performed. Even though validation of these models is challenging due to limited clinical data, *in-vitro* and *ex*-*vivo* experimental data can be utilized to help predict fetal exposure of drugs. PBPK modeling can also serve as a tool to guide drug dosing during breastfeeding based on drug transfer through human milk. In summary, PBPK modeling offers promise as a potential tool to predict maternal and fetal exposure of drugs and thereby guide therapy in this special population.

## Author Contributions

NC: conception or design of the work, acquisition, analysis or interpretation of data for the work, drafting the work, provide approval for publication of the content, and agree to be accountable for all aspects of the work. PD and IS: revising it critically for important intellectual content, provide approval for publication of the content, and agree to be accountable for all aspects of the work. SC and RV: conception or design of the work, revising it critically for important intellectual content, provide approval for publication of the content, and agree to be accountable for all aspects of the work. All authors contributed to the article and approved the submitted version.

## Funding

This work is partially funded by grants from the Eunice Kennedy Shriver National Institute of Child Health and Human Development (NICHD, HD047905) through the Obstetric Pharmacology Research Center (OPRC).

## Conflict of Interest

The authors declare that the research was conducted in the absence of any commercial or financial relationships that could be construed as a potential conflict of interest.

## Publisher's Note

All claims expressed in this article are solely those of the authors and do not necessarily represent those of their affiliated organizations, or those of the publisher, the editors and the reviewers. Any product that may be evaluated in this article, or claim that may be made by its manufacturer, is not guaranteed or endorsed by the publisher.
